# Availability and sufficiency of phenobarbital, an essential medication, in Bhutan: a survey of global and neuropsychiatric relevance

**DOI:** 10.1186/s13104-018-3617-x

**Published:** 2018-08-02

**Authors:** Devender Bhalla

**Affiliations:** 1 Sudan League of epilepsy and Neurology (SLeN), Khartoum, Sudan; 2 Nepal Interest Group of Epilepsy and Neurology (NiGEN), Kathmandu, Nepal; 3 Iran Epilepsy Association, Teheran, Iran

**Keywords:** Anti-epileptic drugs, Epilepsy, Phenobarbital, Treatment gap

## Abstract

**Objective:**

We aimed to provide a reliable evidence-based conclusion around manufacturing, import, availability and sufficiency of one essential medication, phenobarbital (PB) through our example location (Bhutan). The relevant details about manufacturing, import, annual quantity, dose strength were obtained.

**Results:**

There was no local manufacturing of PB and all other anti-seizure medications. A total of 1068 vials of PB 200 mg/mL inj and 489,350 tablets of PB30 mg (i.e. 14.6 kilos) was estimated to annually become available. Of this, 5.3 k (36.3%) was present at the basic health units (BHUs). The PB was absent at 26 (14.7%) BHUs. There was no availability of PB syrup. Treating supposed target of 50.0% of the 20.0% of the prevalent case-load (N = 4523) require 18.1 kilo of PB annually. To conclude, having or not the local manufacturing may or may not be a limitation. There is a need to overcome challenges of inappropriate dose strength, absent pediatric formulation, indirect cost, and low selling price of PB. The possible therapeutic participation of PB in managing disease conditions (like epilepsy) remains limited despite favorable safety and efficacy profile. Strengthening the availability of essential medications is essential to reduce the treatment gap and public health burden of treatable disease conditions.

## Introduction

Phenobarbital (PB) is a recognised essential medication that is indicated for epilepsy and other chronic conditions of global interest, such as anxiety, insomnia, barbiturate addiction, etc. Being essential implies that “*PB satisfies the priority healthcare needs of the population to which there should be access at all times in sufficient quantity (and in a similar manner across regions)*” [1]. Fulfilling this goal is important since patients are likely to die more from treatable conditions (than those which are not) as they may not receive timely or appropriate medication [2]. The risk is even more if those medications are fundamental [3, 4] or are the only ones that are available, feasible, or affordable for the majority of patient population [5–7]. A vital first step to improve access (and therefore coverage) to essential medications is to know their availability in the retail market as well as in the national distribution list. Thus, continuing with our *vision* of establishing a reliable neurological (and mental health) profile and international positive presence of scientifically-silent locations, and with an *objective* to provide reliable conclusions around manufacturing, import, availability and sufficiency of one essential medication (i.e. PB) in our example location (Bhutan), we performed this work. This, if validated, may in-part help to understand access to essential medications and an actual pattern of demand-and-supply before proposing improvements in resource development in neuropsychiatry.

## Main text

### Methods

#### Why Bhutan? What source of medical service? What source of medication?

**Fig. 1:**
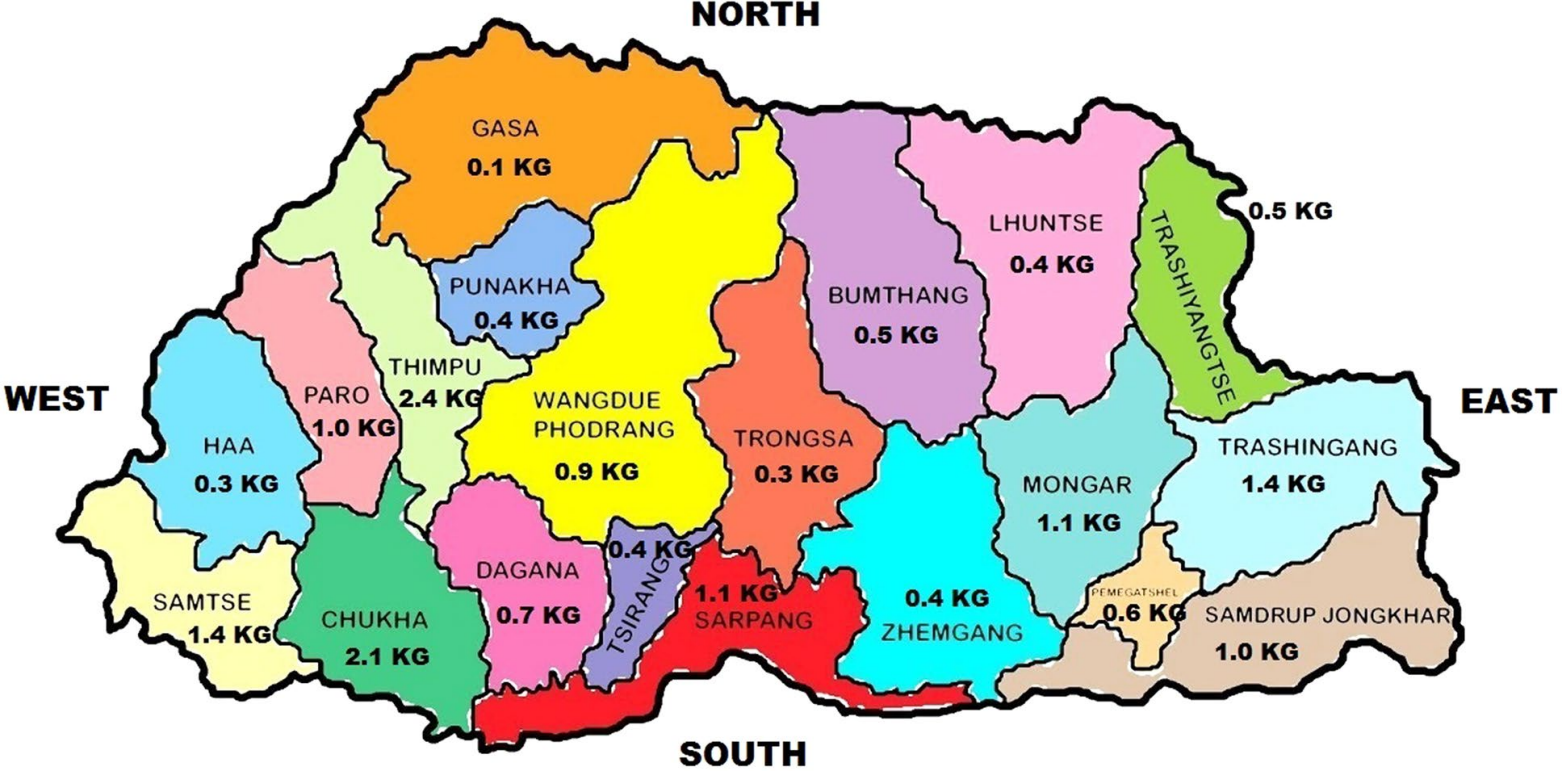
Administrative divisions and depiction of annual in-part requirement of phenobarbital in Bhutan. Kg: the quantity of PB (in kilogram) that is expected to be annually required vis-a-vis prevalent case-load of epilepsy in different divisions of Bhutan

Bhutan is a small landlocked low middle-income Asian country, Fig. 1, with a population of 757,042 (69.1% rural; 52.5% males; density 16.3/KM^2^; 5.9% aged 65+ years; 29.0% aged 0–14 years; life expectancy 71.0 years) [8]. Only 62.0% Bhutan is paved with poor health indicators for those living at the mountainous terrain in comparison to plains [9]. Bhutan has about 185 (average 1 for 4313 residents) basic health units (BHUs). Its healthcare is entirely public with 100% cost-free unconditional medical service [10] although indirect costs (such as time, travel, etc.) remain.

#### What significance of PB (developed and resource-poor countries)?

The relevance of PB differs from person-to-person and region-to-region, depending upon which side one wishes to look at. Generally, PB has a broad anti-seizure (partial and generalised) activity, including neonatal seizures and < 20 min status epilepticus. In resource-poor countries, PB is fundamental, most widely used, and often times the only available or affordable ASM [3, 6, 11]. In developed countries where neurotoxic concerns of PB are reportedly ‘felt’ more, other ASMs have superceded but not necessarily for the right reasons [6, 12, 13]. The position of PB has also been compromised due to its low retail price (low profit margin) and inappropriate marketing of its safety profile. For instance, discontinuation of PB due to actual occurance of side effects was none over 1 year [11] or 1.0% over 2 years [14] or merely 0.6% among children [15]. Thus, supposed greater withdrawal of PB is merely out of this perceived fear [16]. Unlike what is often projected, PB is not an “exclusive” [17], if at all (currently unpublished, D Bhalla 2018), subject of drug abuse [18]. None of the ASMs are superior in efficacy than PB [6, 11, 15] and generally those who do not respond to PB may also not necessarily respond to other ASMs even after four or five regimens [19]. Yet, PB remains “*missing in action*” [20]. One real disadvantage of PB is its low retail price because it leads to little profit margins unlike other ASMs (Field data, D Bhalla 2016) and that in return for severe restrictions and obligations often imposed exclusively [3, 4] on its business [6, 7].

#### Data collection

For *manufacturing*, there was one partially-established pharmaceutical manufacturer. For *import,* all medication (including PB) supplies are imported once per year principally through one centralized department which distribute medicines later-on to all existing health facilities of the country [21] which provided required basis for deriving required estimates on PB for the whole country. For convenience, the scope of this project was limited to PB alone, excluding other ASMs [3]. All data was entered into MS-excel and analysed. The results are presented as count, frequency, mean or median with respective 95% confidence interval (CI) wherever deemed suitable.

Besides this, for epilepsy we analysed sufficiency of PB (i.e. annual availability vis-à-vis annual requirement) for meeting epilepsy-related goals [7, 22] in Bhutan. For this, we assumed that 20.0% of total prevalent epilepsy patient population (PEP) would receive a treatment during first implementation year. Of these 20.0, 50.0% would receive PB and remaining 50.0% would receive other ASMs or other treatments. This PEP was estimated by using an expected epilepsy prevalence of 6.0/1000 [23] and related population data of Bhutan [24]. So, sufficiency was calculated for this scenario as follows:

$$ {\text{Target Population}}\; ( {\text{TP) = ([PEP/5])/2}} $$

$$ {\text{Sufficiency}}\;({\text{S}}) = {\text{ADD}}\;(*)\; 3 6 5\;{\text{days}}\;{\text{of}}\;{\text{year}}\;(*)\;{\text{TP}} $$

An average daily dose (ADD) of PB over an annual treatment period was estimated elsewhere in rural Asia by the author to be 110 mg (per person) to obtain lasting seizure control [11].

### Results

#### Manufacturing and import of PB (frequency, quantity, dosage, administration form)

There was no local pharmaceutical manufacturing of PB (and other ASMs) in Bhutan. For import, during one calendar year (2016–17), a total of 1068 vials of PB 200 mg/mL inj (1 mL vial) and 489,350 ready-tablets of PB 30 mg had been imported. This translates to 14,680,500 mg or 14.6 k of PB. Of this, BHUs received 163 vials (15.2%) of PB inj and 178,200 (36.4%) of PB 30 mg tablets (or 5.3 k of PB). PB was noted to be absent at 26 BHUs (14.7%). No PB pediatric syrup was being manufactured and imported.

#### Epilepsy patient load and sufficiency of PB for the first implementation year

By using an expected epilepsy prevalence of 6.0/1000 [23], about 4536 patients with lifetime epilepsy are present in Bhutan. This number (and relative medication requirement) varies by region, as shown in Table 1. What proportion among them constitutes active epilepsy is not currently known. As explained above on page seven, by taking a lliberal scenario of treating 50.0% of the 20.0% prevalent epilepsy patients with PB (i.e. N = [4536/5]/2), the annual requirement of PB for epilepsy during first implementation year then comes at 18.1 k. This requirement during subsequent years may change depending principally upon how many move in and out of this target population pool, for each evaluation period.

**Table 1:** People with epilepsy and annual in-part requirement of PB across different divisions of Bhutan

Province	Population	PEP	Target pop	PB requirement/year (Kg)	3 Km (%)	P/D
Bumthang	18,965	114	11	0.5	75	7715
Chhukha	88,342	530	53	2.1	NA	4908
Dagana	27,522	165	16	0.7	NA	NA
Gasa	3,694	22	2	0.1	NA	3694
Haa	13,499	81	8	0.3	71	1227
Lhuentse	17,618	106	11	0.4	87	17,201
Monggar	44,259	266	27	1.1	89	2168
Paro	43,167	259	26	1.0	NA	10,415
Pemagatshel	25,176	151	15	0.6	68	8394
Punakha	17,715	106	11	0.4	NA	NA
Samdrupjonkhar	39,961	240	24	1.0	NA	NA
Samtse	60,100	361	36	1.4	NA	NA
Sarpang	45,637	274	27	1.1	93	2985
Thimphu	98,676	592	59	2.4	94	1015
Trashigang	56,168	337	34	1.4	NA	9361
Trashiyangtse	20,874	125	12	0.5	72	5139
Trongsa	13,419	81	8	0.3	NA	NA
Tsirang	18,667	112	11	0.4	NA	NA
Wangdue	36,922	222	22	0.9	NA	NA
Zhemgang	18,636	112	11	0.4	NA	NA

Annual requirement of PB100 mg tablets is based on prevalent lifetime epilepsy pool of patients. Target population was defined as treating 50.0% of the 20.0% of the prevalent epilepsy population (i.e. column 4)

*3Km* proportion of population within 3 Km walking distance, *NA* no information available, *PEP* prevalent epilepsy population, *PB* phenobarbital, *P/D* number of persons per doctor

### Discussion

In Bhutan, there is no fully-established local pharmaceutical manufacturing and medicine supplies here depend entirely on import. So, the first question may include the pros and cons of local pharmaceutical manufacturing. In other rural countries, like Cambodia, the health department produces its own PB brand (Field data, D Bhalla 2010). Elsewhere, while some prefer small countries like Bhutan to rely on import for [25], others suggest establishing a local pharmaceutical industry [26] as it may provide direct positive impact on the country’s economic development [27]. There may not be any clearcut answer to this but, whether desired or not, the pharmaceutical import remains largely a necessity. For instance, 90% of the medicines available in sub-Saharan Africa are imported from outside [26]. This is not unexpected since raw material is no-to-rarely available, as it is here and in neighbouring Asia (Field data, D Bhalla 2016).

We estimated that about 15 kilos of PB became available over an annual period. Thus, in comparison to African countries, Bhutan is doing a better job to make speciality essential medications available in the country, at least to some extent. For instance, in 14 African countries, a sub-set of 20 basic essential medicines were found to severely lack in their availability at all levels of healthcare and distribution [28]. In such a scenario where ready (and essential) solutions are not sufficiently becoming available for the patients, we may not expect to bridge our goals related to reducing the treatment gap and public health burden of treatable conditions. Not surprisingly, PB is formally recognised as “*missing in action*” despite having a favorable efficacy and safety profile [20].

The reason that PB gets freely imported here is that all healthcare functions are predominantly public in nature. This differs from other countries where public system is not strong and sufficient and private agencies are subjected to restrictions [6, 7] (Field data, D Bhalla, 2016). Such restrictions are seen to push private stakeholders towards “easier” substances and away from the business of PB [29]. Few ministries elsewhere have realised the need of engaging private agencies to meet their healthcare goals for their own benefit and of their patients [30]; which Bhutan has yet to learn. Few ministries have also had their epilepsy control programs based principally on PB [31, 32].

Here, PB was available in 30 mg dose strength, which certainly require patients to take multiple tablets at a single time, several times a day. This is an important factor towards inconvenience and non-adherence [11]. Similarly, pediatric PB syrups were absent as in other countries like Brazil, Nepal [3] (Field data, D Bhalla, 2016), an important system-related deficiency.

Here, about 36.0% of PB was at BHUs i.e. supposedly ‘nearest’ to the population, Table 1. On the positive side, this is a better situation than elsewhere, for instance in 14 African countries, basic essential medications were available at 18–48.0% of primary health centers alone [28]. On the negative side, being ‘nearest’ may have a different context here since only 62.0% of this country is paved. Although the target is to keep the whole population within 3 Km walking distance [33], this would still not be an ideal situation [34, 35]. Lastly, as is the case with PB here, complex neuropsychiatric topics are often projected through negativity and continual transfer of by-default opinions from one to another, as the author has shown for Asia, Africa, Middle-east and North Africa, South America, The Caribbean [36], causing undue fear, negativity, and erroneous conclusions.

Our topic was suitable, of immense need and relevance, and was addressed through one scientifically-silent country [10] by using a simple replicable method. The topic has multiple dimensions as shown above. Our work also helped to advance global goals and recommendations such as the need for promotion of research and education on epilepsy, publication of detailed public health assessments etc. [22]. Our work also helps to focus on better use of “ready solutions” of which many patients may remain largely un-benefitted [6, 11, 31, 32].

To conclude, Bhutan does not manufacture PB (and all other ASMs) and annually makes available about 15 kilos of PB. This mainly happens because of the public nature of all healthcare functions here. In terms of sufficiency and making available specialised essential medications, Bhutan is doing a better job than many African countries. However, the possible therapeutic participation of PB in managing disease conditions (like epilepsy) likely to remain limited despite favorable safety and efficacy profile. There is a need to strengthen the availability of essential medications to reduce the treatment gap and public health burden of treatable disease conditions through fuller use of ready solutions. There is also a need to overcome challenges of inappropriate dose strength, absent pediatric formulation, indirect cost, and low selling price of PB.

## Limitations

The limitations may include that we restricted ourselves, out of convenience and complexity, to PB alone although many other ASMs (including diazepam, phenytoin, sodium valproate) are possibly available. However, the question here was not just what is available but how much is available, and what level of treatment coverage may possibly be achieved with current quantity of one essential, affordable, and feasible medication. We currently do not know what proportion of patients are possibly treated with each ASMs in Bhutan, which is an important future question. The sufficiency projection may vary, but not our principal result about the quantity, depending upon how many are to be treated with PB. Our work did also not take into account projections for other disease conditions where PB is indicated, and also for the requirement due to incident cases and duration beyond 1 year.

## Abbreviations

ADD: average daily dose
ASM: anti-seizure medication
BHU: basic health unit
PB: phenobarbital
PEP: prevalent epilepsy patient population
